# Treatment of true superficial femoral artery aneurysms: the 15-year experience of a single centre

**DOI:** 10.1308/rcsann.2023.0078

**Published:** 2023-11-20

**Authors:** G Zenunaj, G Baldazzi, P Acciarri, V Gasbarro, AM Cosacco, R Serra, L Traina

**Affiliations:** ^1^ University Hospital of Ferrara, Italy; ^2^ University of Ferrara, Italy; ^3^ Università Magna Graecia di Catanzaro, Italy

**Keywords:** Femoral artery, Femoral aneurysm, Endovascular repair, Peripheral aneurysm

## Abstract

**Objective:**

True superficial femoral artery aneurysms (SFAAs) are rare and traditionally treated by open repair. However, the endovascular approach excluding the aneurysm sac with a covered stent may be an alternative. This study aimed to compare the outcomes of the open and endovascular repair of SFAAs.

**Methods:**

This is a retrospective, observational, monocentric study. The main endpoints were: technical success, limb salvage and primary patency rate, and hospitalisation time.

**Results:**

We identified 49 SFAAs in 40 patients; the mean age was 73.3±10.1 years, the mean diameter of SFAAs was 5.41±3.64cm, and 61.2% were symptomatic for ischaemic or compression-related signs. The indication for open repair was given mainly for complex SFAAs involving the distal third of the superficial femoral artery and with an ipsilateral popliteal aneurysm. Among the 36 open-repair patients, 33 underwent ligation and revascularisation via bypass or graft interposition, and 3 patients underwent simple ligation without revascularisation. The endovascular approach was adopted mainly for aneurysms located in the medial third of the SFAA, which underwent covered stenting in 12 patients and coil embolisation in 1 patient. The technical success was 100% in all cases. There were no statistical differences in terms of primary patency and limb salvage rate between groups at two and four years. The mean hospitalisation time was 10±4 and 3±1 days after open and endovascular treatment, respectively.

**Conclusions:**

The endovascular approach may be a valid alternative for isolating SFAAs offering good results and shorter hospitalisation. Open repair remains a valid approach, particularly in complex aneurysms.

## Introduction

True superficial femoral artery aneurysms (SFAAs) are rare because most cases are diagnosed late in life. Late diagnosis is usually related to the difficulty in recognising them earlier because they are located deep in the thigh and are not evident during physical examination unless they present as a painful pulsatile mass. Consequently, they continue to grow for many years until they reach considerable diameters and become symptomatic, mainly with symptoms related to mass compression of the adjacent vein and nerve structures. Moreover, non-mass-related symptoms include distal embolisation and rupture, which increase the risk of limb loss.^1^ Open surgery has been the mainstay of treatment of SFAAs and involves mass decompression by opening the aneurysm sac followed by revascularisation with a prosthetic or vein graft.^2,3^ The endovascular approach has recently gained popularity and is expected to become the preferred choice in the future. In the current study, we report our 15 years of experience in treating SFAAs, with a focus on the outcomes of patients undergoing open repair versus endovascular repair.

## Methods

This was a retrospective, observational, single-centre study. We analysed our institutional electronic database (Ormaweb O4C, Dedalus, Firenze, Italy) to identify all patients who underwent surgical repair for true SFAA with either open or endovascular approach between January 2005 and March 2021. The search was performed using the 442.3 ICD-9 code, which refers to lower limb aneurysms, and then we selected only the SFAAs for the study. Paper-based and electronic medical records were reviewed with regard to their demographics and comorbidities.

The primary endpoints were technical success, which is defined as the absence of any perioperative complications related to limb and cardiovascular events; limb salvage, which is defined as the absence of major limb amputation during the follow-up; and primary patency, which is defined as the absence of restenosis >40% or peak systolic flow >150m/s on ultrasound at the graft and anastomosis in the open group and at the landing zones of the stent graft in the endovascular group. The secondary endpoints included major adverse cardiac events (MACE), survival and hospitalisation time. Follow-up consisted of clinical and ultrasound examinations at one, three and six months and annually thereafter.

### Data analysis

Records of all patients were collected and tabulated in Microsoft Excel (Microsoft Corporation, Redmond, WA, USA). The Kaplan–Meier life table method was used to assess primary patency, limb salvage and patient survival via MedCalc 20.35. Chi-square test and Fisher’s exact test were used to compare the categoric variables responsible for the outcomes. *p*<0.05 was considered significant.

This study was approved by the Institutional Review Board of Independent Ethics Committee and registered with N.ER.FE.2018.66. Written consent from patients for the publication of images in this paper was obtained and is available if required.

## Results

We identified 49 SFAAs in 40 patients treated between January 2005 and March 2021. Approximately 90% of the SFAAs were identified in men (36/40). The mean age at the time of diagnosis was 73.3±10.1 years. The demographic data and comorbidities are presented in Table 1.

**Table 1:** Patients’ demographic and clinical data and aneurysm presentation

Variables	*N* (%)	Open, *N* (%)	Endo, *N* (%)	*p*
Patients	40 (100)	30 (75)	10 (25)	
Age, years (mean±SD)	73.3±10.1	73±10.2	75.4±9.9	
Male sex	36 (90)	28 (93.3)	8 (80)	0.223
Comorbidities				
Active smoking	15 (37.5)	10 (33.3)	5 (50)	0.345
Hypertension	25 (62.5)	20 (66.7)	5 (50)	0.345
Coronary artery disease	8 (20)	5 (16.7)	3 (30)	0.361
Nonischemic cardiopathy	13 (32.5)	10 (33.3)	3 (30)	0.845
Arrhythmia	15 (37.5)	11 (36.7)	4 (40)	0.850
Atrial fibrillation	6 (15)	5 (16.6)	1 (10)	
Diabetes	4 (10)	4 (13.3)	0	0.223
Chronic kidney disease	9 (22.5)	5 (16.7)	2 (20)	0.810
Dyslipidemia	16 (40)	10 (33.3)	6 (60)	0.136
Previous stroke	3 (7.5)	3 (10)	0	0.078

On admission, 31 (77.5%) patients presented with a history of aneurysm or current aneurysm in another anatomical region (see Table 2). Most SFAAs were located in the middle and distal thirds. The mean diameter was 5.41±3.64cm (2.7–15.5cm). Overall, 30 (61.2%) patients with SFAAs were symptomatic; 3 presented with ruptures, and 6 had signs of limb ischaemia due to thrombosis (4 cases) and distal embolisation (2 cases). Most of the SFAAs (21 cases) presented with symptoms due to “mass effect”, such as leg swelling and pain pulsatile mass. Finally, 19 patients with SFAAs were asymptomatic and referred to our institution after instrumental examinations were performed for other medical conditions. Ultrasonography was the first instrumental examination in all symptomatic SFAA cases and in ten asymptomatic cases. Computed tomography (CT) of the aorta, iliac arteries and arteries of the lower limb was performed to better evaluate the SFAA and identify other aneurysms. The remaining nine asymptomatic SFAAs were discovered by CT scan in eight cases, and magnetic resonance imaging was performed for other medical conditions.

**Table 2:** Clinical and anatomical presentation of SFAAs

	*N* (%)	Open, *N* (%)	Endo, *N* (%)	*p*
Patients	40 (100)	30	10	
Diameter, cm (mean±SD)	5.41±3.64	5.57±4.07	4.55±3.4	
Aneurysms elsewhere	31 (77.5)	25	6	0.125
Aortic aneurysms	12 (38.7)	11 (44)	1 (16.7)	0.111
Iliac aneurysms	9 (29)	8 (32)	1 (16.7)	0.274
Ipsilateral CFAA	5 (16.1)	5 (20)	0	0.167
Contralateral SFA	3 (9.7)	2 (8)	1 (16.7)	0.728
Ipsilateral PAA	15 (48.4)	12 (48)	3 (50)	0.571
Limbs with SFAA	49 (100)	36	13	
Localisation in the SFA				
Proximal segment	7 (14.3)	6 (16.7)	1 (7.7)	0.428
Middle segment	17 (34.7)	9 (25)	8 (61.5)	0.017
Distal segment	9 (18.4)	8 (22.2)	1 (7.7)	0.246
Middle and distal segments	10 (20.4)	8 (22.2)	2 (15.4)	0.600
Whole SFA	3 (6.1)	3 (8.3)	0 (0)	0.282
Not reported	3 (6.1)	2 (5.6)	0 (0)	0.385
Ipsilateral PAA	15 (30.6)	12 (33.3)	3 (23)	0.571
Symptoms	30 (61.2)	21	9	
Compression	8 (26.7)	6 (28.6)	2 (22.2)	0.914
Painful pulsatile mass	13 (43.3)	9 (42.9)	4 (44.4)	0.686
Ischaemic-related signs	6 (20)	4 (19.1)	2 (22.2)	0.687
Rupture	3 (10)	2 (9.5)	1 (11.1)	0.782

cFAA = common femoral artery aneurysm; PAA = popliteal artery aneurysm; SFA = superficial femoral artery; SFAA = superficial femoral artery aneurysm

### Surgical management

The open group consisted of 36 patients with aneurysm, and 33 of these patients underwent ligation, aneurysm decompression and subsequent revascularisation via bypass or graft interposition. Among the 16 bypass procedures, 7 were performed using the great saphenous vein (GSV), 7 were performed using an expanded polytetrafluoroethylene (ePTFE) prosthesis and 2 were performed using a composite graft (ePTFE and vein). By contrast, graft interposition was performed in 17 cases: GSV, ePTFE and polyester were used in 5, 9 and 3 cases, respectively. Finally, three cases underwent simple ligation without subsequent revascularisation. These patients were followed up in the outpatient clinic every three months for the first year and then annually, and no intervention was needed. Before surgery five patients were taking anticoagulant therapy for cardiac indication, which was maintained after the revascularisation. The remaining patients were set up with lifelong single antiplatelet therapy.

The endovascular approach was indicated mainly for aneurysms located in the middle third of the SFA (0.017). In the endovascular group, all procedures were performed using an antegrade ipsilateral approach, and 12 SFAAs were excluded percutaneously by deploying a covered stent graft (Viabahn, Gore Excluder) under local anaesthesia in eight cases and with superficial femoral artery cutdown in the four remaining SFAA cases. For percutaneous procedures, an 8 Fr short sheath was used, and haemostasis was performed using a percutaneous closure device (AngioSeal Vip 8 Fr, Terumo Medical Corporation, Somerset, NJ, USA). In one case with ruptured SFAA, the aneurysm sac was embolised by coils (16 cases with 8mm embolisation coils, MReye® Embolisation Coil, Cook Medical, Bloomington, USA).^4^ This decision was made because the patient had a history of a popliteal prosthetic graft for ipsilateral popliteal artery aneurysm (PAA), which at the CT scan was occluded in the absence of limb ischaemic signs. According to our internal protocol, patients underwent dual antiplatelet therapy (aspirin 100mg and clopidogrel 75mg) for the first three months and then were maintained under lifelong single antiplatelet therapy daily (aspirin 100mg). One patient was under anticoagulant therapy for atrial fibrillation before surgery, and after the procedure aspirin 100mg was introduced for the first three months and then followed only with the anticoagulation. The patient who underwent coil embolisation maintained the single antiplatelet therapy as he had a history for CAD.

### Postoperative results and follow-up

The technical success rate was 100% in all cases. The mean hospitalisation time was 10±4 and 3±1 days after open surgery and endovascular revascularisation (*p*<0.001), respectively. In the open group, five patients developed surgical site haematoma, with two patients requiring revision and drainage. No other surgical site complications occurred until hospital discharge.

**Figure 1:**
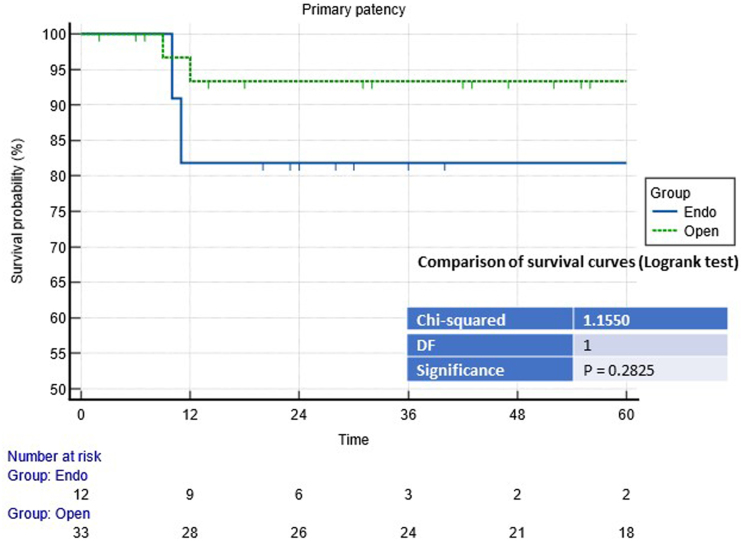
Comparison of survival curves (Logrank test) for the primary patency rate

The mean follow-up period was 60 months (range 24–204 months). The primary patency rates in the open group at one and five years was 93.7% and 81.2%, respectively, whereas that in the endovascular group was 83% at one and five years; there were no statistical differences between the two groups (see Figure 1). In the endovascular group, stent graft occlusion occurred in two patients with concomitant PAA, both of whom underwent open repair by using the GSV as a graft.

**Figure 2:**
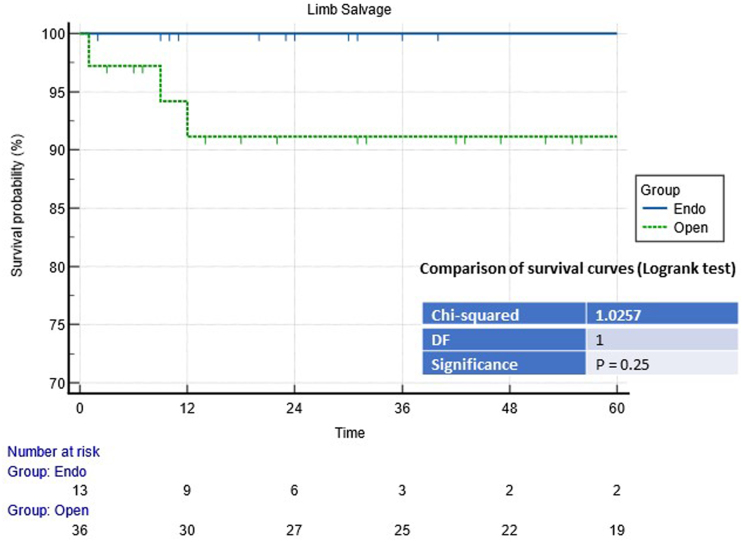
Comparison of survival curves (Logrank test) for the limb salvage rate

The limb salvage rate was 90.7% and 100% after 12 months in the open and endovascular groups, respectively (*p*=0.25) (see Figure 2). In the open group, one patient who underwent revascularisation with ePTFE graft interposition for symptomatic SFAA developed reperfusion syndrome with compartment limb and systemic presentation and required urgent knee amputation five days after revascularisation. During follow-up, the other two patients underwent knee amputation for severe ischaemia due to prosthetic graft occlusion.

**Figure 3:**
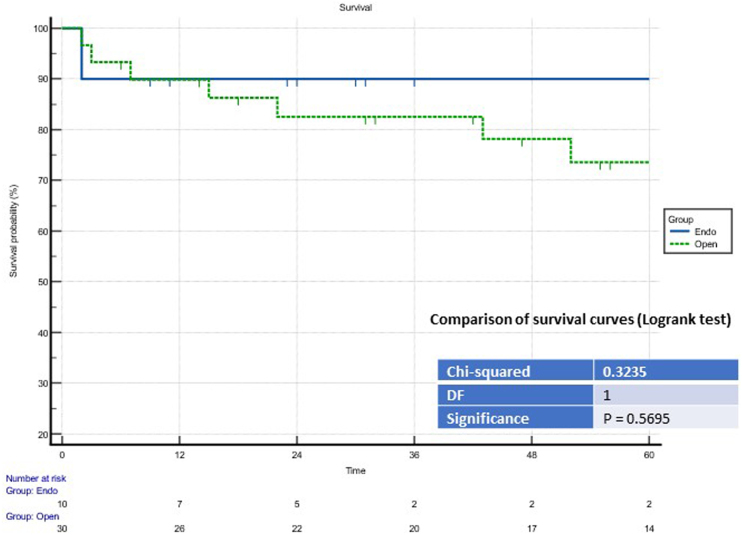
Comparison of survival curves (Logrank test) for the survival rate

**Figure 4:**
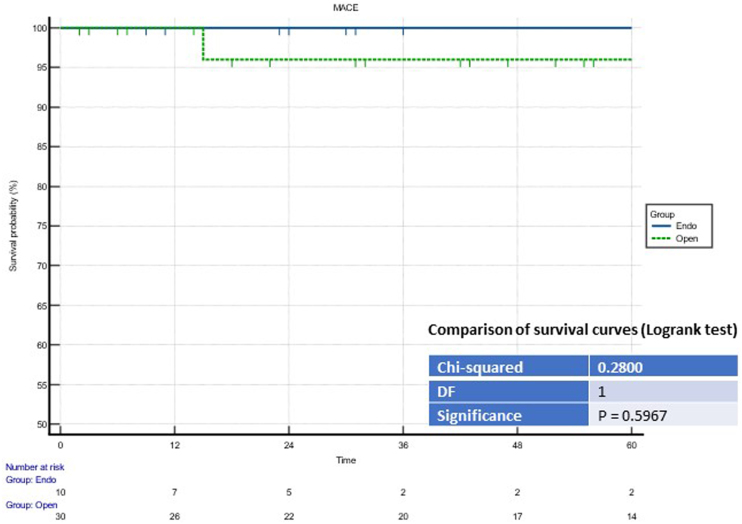
Comparison of survival curves (Logrank test) for the major adverse cardiovascular events (MACE)

No periprocedural or 30-day deaths occurred. At five years, there were no statistically significant differences in the MACE and survival rates (see Figures 3 and 4).

## Discussion

SFAAs are rarely reported because these aneurysms often remain asymptomatic and continue to grow until they reach a considerable size. They present mainly with symptoms of mass compression of the adjacent vein and nerve structures and rarely with rupture.^4^ The remaining cases presented with signs of lower limb ischaemia due to thrombosis of the aneurysm sac or distal embolisation, thus increasing the risk of limb loss; this situation was independent of the aneurysm diameter.^1–3^ Most studies consist of case reports, and few papers have reported a case series. We report one of the largest case series from a single centre and compared the outcomes of open and endovascular approaches for true SFAAs. In our series, the clinical limb presentation and location of the SFAA did not differ significantly from those reported in the current literature.^5^ As reported in the guidelines, there is a high consensus for the repair of symptomatic SFAAs to remove the embolic source, eliminate any mass effect and restore limb perfusion.^6^ By contrast, there is still no consensus on the treatment for asymptomatic cases.^1^ Several surgical approaches have been described in the literature; however, the open approach remains the mainstay of treatment and has been used in almost 70% of all cases reported in the literature and in 73.4% of cases in our experience. These approaches include several techniques, such as aneurysm ligation without revascularisation, especially in patients with high surgical risk (literature vs our centre was 7.3% and 6.1%, respectively); primary amputation (rarely applied in 3% of cases); and aneurysmectomy with revascularisation using a prosthetic or vein graft. In both our series and the literature, prosthetic material, particularly ePTFE, represents the graft material that was most utilised for reconstructions, and an interposition configuration rather than a bypass was preferred.

Endovascular treatment has gained popularity in the last five years, accounting for 52.6% of SFAAs reported in the literature^5,7^ and representing 26.5% of cases in our series. Our study demonstrated a significant difference in in-hospital stay compared with the open-to-endovascular approach. This is an expected outcome considering that most of the procedures are performed under local anaesthesia and percutaneously or by the femoral artery cutdown technique, which has been demonstrated to be a safe and less invasive approach that results in less postoperative pain and faster mobilisation.^8^ For these reasons, this minimally invasive approach is advantageous, especially in the elderly and frail patients as in our case series. However, in the present study, we did not notice any statistical differences in mid- and long-term survival or MACE rates between the two groups.

Perini *et al*^2^ reported one of the largest case series with a five-year follow-up period and an estimated five-year graft patency rate of 85% after open repair. In our series, the primary patency rate in the open group was 93.7% after one year and 81.2% after five years. In the endovascular group, the primary patency rate was lower at 12 months but was not statistically significant (*p*=0.2). The number of cases treated with the endovascular approach is too small to draw solid conclusions. However, as in our experience, there was a strong selection of cases undergoing this kind of treatment, thus resulting in SFAAs mainly located in the middle third of the SFA. In the open group, there were more complex aneurysms in the distal third of the SFA and both the middle and distal thirds of the SFAA. The involvement of the popliteal artery was the most important factor in 12 cases. Moreover, the two stent graft thromboses in the endovascular group occurred in two patients who presented with PAA. In the remaining cases, primary patency was 100% during the entire follow-up period. The risk of stent graft occlusion after endovascular treatment of PAAs is high, is well described in the literature, and is related mainly to forces exerted on the stent during knee flexion, which leads to continuous stress on the stent graft structure.^9–11^ Considering this aspect, open revascularisation in our experience demonstrated excellent results in complex SFAAs in the long term regarding primary patency and limb salvage rates.

The main limitation of this study was its small sample size, which was related to the rare frequency of these aneurysms. The second limitation is related to the retrospective nature of the study, which led to a bias in offering an endovascular approach for aneurysms located mainly in the middle third of the SFA.

## Conclusions

The endovascular approach is becoming increasingly popular and could be a valid alternative for isolated SFAAs. It offers similar primary patency and limb salvage as open surgery and short hospitalisation times. By contrast, open revascularisation remains a valid approach, particularly for complex SFAAs and ipsilateral PAA.

## Author contributions

Dr Gladiol Zenunaj and Luca Traina share the first authorship. Conception and design: GZ, Analysis and interpretation: AC, GB. Data collection: AMC, GB. Writing the article: LT, GZ. Critical revision of the article: VG, RS. Final approval: VG. -Overall responsibility: VG, LT.
